# WISP1 induces the expression of macrophage migration inhibitory factor in human lung fibroblasts through Src kinases and EGFR-activated signaling pathways

**DOI:** 10.1152/ajpcell.00410.2023

**Published:** 2023-12-25

**Authors:** Maria-Elpida Christopoulou, Spyros S. Skandalis, Eleni Papakonstantinou, Daiana Stolz, Alexios J. Aletras

**Affiliations:** ^1^Laboratory of Biochemistry, Department of Chemistry, University of Patras, Patras, Greece; ^2^Clinic of Pneumology, Medical Center-University of Freiburg, Faculty of Medicine, University of Freiburg, Freiburg, Germany

**Keywords:** lung inflammation, WISP1/CCN4, MIF, EGFR, Src kinases

## Abstract

Wnt1-inducible signaling protein 1 (WISP1/CCN4) is a secreted matricellular protein that is implicated in lung and airway remodeling. The macrophage migration inhibitory factor (MIF) is a pleiotropic cytokine that has been associated with chronic lung diseases. In this study, we aimed to investigate the WISP1 signaling pathway and its ability to induce the expression of MIF in primary cultures of fibroblasts from normal human lungs (HLFs). Our results showed that WISP1 significantly stimulated the expression of MIF in a concentration- and time-dependent fashion. In WISP1-induced expression of MIF, αvβ5-integrin and chondroitin sulfate proteoglycans as well as Src tyrosine kinases, MAP kinases, phosphatidylinositol 3-kinase/Akt, PKC, and NF-κB were involved. WISP1-induced expression of MIF was attenuated in the presence of the Src kinase inhibitor PP2 or the MIF tautomerase activity inhibitor ISO-1. Moreover, WISP1 significantly increased the phosphorylation and activation of EGF receptor (EGFR) through transactivation by Src kinases. WISP1 also induced the expression of MIF receptor CD74 and coreceptor CD44, through which MIF exerts its effects on HLFs. In addition, it was found that MIF induced its own expression, as well as its receptors CD74/CD44, acting in an autocrine manner. Finally, WISP1-induced MIF promoted the expression of cyclooxygenase 2, prostaglandin E_2_, IL-6, and matrix metalloproteinase-2 demonstrating the regulatory role of WISP1-MIF axis in lung inflammation and remodeling involving mainly integrin αvβ5, Src kinases, PKC, NF-κB, and EGFR. The specific signaling pathways involved in WISP1-induced expression of MIF may prove to be excellent candidates for novel targets to control inflammation in chronic lung diseases.

**NEW & NOTEWORTHY** The present study demonstrates for the first time that Wnt1-inducible signaling protein 1 (WISP1) regulates migration inhibitory factor (MIF) expression and activity and identifies the main signaling pathways involved. The newly discovered WISP1-MIF axis may drive lung inflammation and could result in the design of novel targeted therapies in inflammatory lung diseases.

## Graphical abstract

**Figure:**
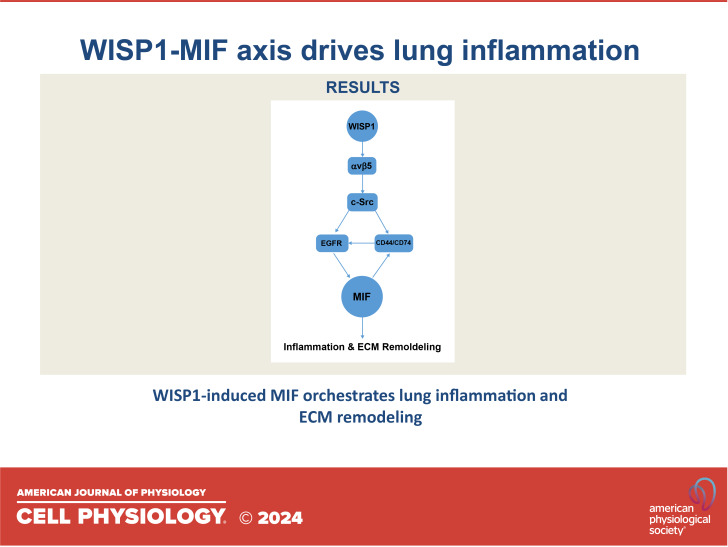

## INTRODUCTION

WNT-inducible signaling pathway protein-1 (WISP1; also known as CCN4) is a secreted, matricellular protein that is assigned to the CCN protein family (1, 2). As a matricellular protein, WISP1 does not interact with specific membrane receptors but rather with integrins to regulate intracellular signaling molecules and pathways (3, 4), including c-Src kinases, RAS/RAF/MEK/ERK, JNK/p38, NF-κB, and phosphatidylinositol 3-kinase (PI3K)/Akt (5–10). Consequently, it is implicated in several processes such as cell adhesion, proliferation, differentiation, survival, motility, angiogenesis, and wound healing/tissue repair (11, 12). Numerous studies have documented that WISP1 or WNT signaling abnormalities are associated with many respiratory diseases (13–18). WISP1 is involved in airway remodeling in asthma by inducing fibroblast proliferation and extracellular matrix (ECM) deposition through activation of Akt/GSK-3β signaling (16, 17, 19–23). Moreover, WISP1 has been found to be increased in alveolar epithelium type II cells in patients with idiopathic pulmonary fibrosis (IPF) and correlates with (myo)fibroblast activation, ECM deposition, and epithelial-mesenchymal transition (13–15). Furthermore, it has been reported that β-catenin signaling contributes to ECM production by lung fibroblasts and their differentiation to myofibroblasts in chronic obstructive pulmonary disease (COPD) (24, 25). On the other hand, suppressed Wnt/β-catenin signaling has been suggested to play an essential role in airway inflammation in COPD by promoting the production of inflammatory cytokines via a peroxisome proliferator-activated receptor-δ/p38 signaling pathway in airway epithelial cells (26). Thus, the precise role of the WNT/β-catenin pathway and WISP1 in COPD remains unclear and requires further investigation.

Macrophage migration inhibitory factor (MIF) is a proinflammatory mediator that plays an important role in the innate immune response. It is expressed in various cell types, such as monocytes/macrophages, T lymphocytes, eosinophils, and epithelial and endothelial cells, after stimulation with inflammatory molecules or exposure to stress (27). MIF can bind to cell surface receptors, such as CD74 and its coreceptor CD44 and CXCRs, or enter cells via endocytosis (28–30). Although both CD74 and CD44 receptors lack intrinsic kinase activity, the coreceptor CD44 is known to interact with Src kinases, which in turn can activate the RAS/RAF/MEK1/2/ERK1/2 signaling pathway (31–33) and, subsequently, several transcription factors including c-myc, NF-κB, and Ets. MIF is also able to induce NF-κB activation through its interaction with thioredoxin-interacting protein (TXNIP), a tumor suppressor and known inhibitor of NF-κB activity (34).

MIF possesses diverse catalytic activities such as tautomerase (35, 36) and thiolprotein oxidoreductase (37) enzymatic activity. Several functions of MIF have been linked to its unusual enzymatic properties (38–41). Various compounds able to specifically inhibit the tautomerase activity of MIF have been synthesized, among them the (S,R)-3-(4-hydroxyphenyl)-4,5-dihydro-5-isoxazole acetic acid methyl ester (ISO-1) (38, 42). ISO-1 is able to suppress several biological activities of MIF, such as the MIF-induced expression of TNF-α, INF-γ, IL-4, IL-17, COX-2, and prostaglandin E_2_ (PGE_2_) production (42).

MIF has been associated with inflammatory and interstitial lung diseases, including COPD, asthma, and IPF (43). Its levels have been found to be increased in bronchoalveolar lavage (BAL) fluid, serum, and sputum from asthma patients (44). In addition, MIF promotes the proliferation and migration of airway smooth muscle cells (ASMCs) by triggering ERK1/2 and FAK signaling pathways (45) and may enhance airway remodeling in asthma by promoting autophagy of ASMCs (46). Furthermore, MIF-deficient mice showed reduced lung inflammation and airway hyperresponsiveness in an ovalbumin-induced asthma model (47), while ISO-1 inhibited airway remodeling in the same model (48). In COPD, MIF induces cell proliferation and protects cells from death. MIF can protect from emphysema and promotes a prorepair response, which may contribute to bronchitis and airway remodeling. In addition, the levels of MIF in the BAL of IPF patients were higher than in control subjects (49). Although WISP1 and MIF are present in lung tissue and both play a role in the development of various lung diseases, their interrelationship has not been established yet. In this study, we demonstrate for first time that WISP1 is capable of inducing the expression of MIF in lung fibroblasts. Furthermore, we provide insights into the molecular mechanisms that underlie this phenomenon.

## MATERIALS AND METHODS

### Materials

U0126, SP600125, LY294002, Bay-11-7085, forskolin, phorbol-12-myristate-13-acetate (PMA), 2′,5′-dideoxy adenosine, Ro31-8220, AG-1478, H-89, genistein, collagenase from *Clostridium histollyticum* (Sigma-Aldrich Chemie, Taufkirchen, Germany), 3-(4,5-dmethylthiazol-2-yl)-2,5-diphenyltetrazolium bromide (MTT), and anti-α-tubulin rabbit polyclonal antibody were purchased from Sigma-Aldrich (St. Louis, MO); PP2 and SB203580 were from Tocris Biosience (Bio-Techne Ltd, Abingdon Oxon UK); leupeptin was from Roche (Switzerland); and protein A agarose suspension was from Millipore (Merk Group, Darmstadt, Germany). ISO-1 was a gift of Dr. Al-Abed, Y. (The Picower Institute for Medical Research, Manhasset, NY). Chondroitinase ABC and AC were purchased from Seikagaku Corporation (Japan). DMEM low glucose, Dulbecco’s PBS (DPBS), and dimethylsulfoxide (DMSO) were purchased from PAN-Biotech (Germany), and trypsin-EDTA, penicillin/streptomycin, and fetal bovine serum (FBS) were rom BioSera (France). Recombinant human WISP1, endotoxin free, and MIF active were purchased from PeproTech (Rocky Hill, NJ) and ImmunoTools (Friesoythe Germany), respectively. Recombinant human EGF and anti-integrin αvβ5 mouse mAb (no. MAB2528-SP) were purchased from R&D Systems (Bio-Techne Ltd., Abingdon, UK). Horseradish peroxidase (HRP)-conjugated streptavidin, anti-P-Tyr mouse mAb (no. 309302), and purified mouse IgG_1_ (no. 400101) were purchased from BioLegend (San Diego, CA). Antibodies against Akt (40D4, no. 2920) and P-Akt (Ser473; D9EXP, no. 4060), NF-κB p65 (D14E12), and P-NF-κB p65 (Ser536; 93H1), Erk1/2 (no. 9102), and P-Erk1/2 (Thr202/Tyr204, no. 9101) were purchased from Cell Signaling (Danvers, Massachusetts). Anti-EGF receptor (anti-EGFR) rabbit mAb (no. NBP2-52651) and polyclonal (no. NBP2-92965, specific for immunoprecipitation) antibodies were purchased from Novus (Bio-Techne Ltd., Abingdon Oxon UK). Anti-CD74 mouse mAb (no. 66390-1-Ig) was purchased from Proteintech (Planegg-Martinsried, Germany). Anti-CD44 mouse mAb (Hermes-3) was kindly provided by Dr. S. Jalkanen (University of Turku, Finland). 1-Step Ultra TMB-ELISA and Immobilon Crescendo Western HRP Substrate were purchased from Thermo Scientific (Waltham MA) and Millipore (Merk Group, Darmstadt, Germany), respectively. All other chemicals were of analytical grade and purchased from Sigma-Aldrich (St. Louis, MO).

### Isolation and Culture of Primary Human Lung Fibroblasts

Human lung fibroblasts (HLFs) were isolated from the adjacent normal region of human lung tumor resection obtained from three donors (*n* = 3; 2 males and 1 female). The lung samples were minced and digested with *Clostridium* collagenase type II (1 mg/mL) in serum-free Dulbecco’s modified Eagle’s medium (DMEM) at 37°C for 2 h. Samples were then filtered through nylon filters, and the filtrates containing the cells were centrifuged at 1,700 rpm for 4 min. The pellets were washed with DMEM, resuspended in DMEM supplemented with 10% heat-inactivated fetal bovine serum (FBS) and 1% penicillin and streptomycin (P/S), plated on 75-cm^2^ cell culture flasks, and grown in a humidified atmosphere with 5% CO_2_. After overnight culture, the nonadherent cells were removed and the adherent cells were cultured in DMEM plus 10% FBS and 1% P/S. At 80–90% confluence, cells were trypsinized, divided 1:3, and recultured in the same medium. For all experiments, cells were used in passages over four. HLFs were seeded on six-well plates and cultured until confluent. Then, they were starved in serum-free DMEM for 24 h before the experiments. All studies were conducted on primary cells from each donor (*n* = 3), and the results were obtained from three independent experiments. The various factors/inhibitors used in the present study were dissolved in DMSO, and therefore, the respective controls contained the same concentration of DMSO.

The viability of cells against several inhibitors used in this study was examined by the MTT assay. None of the inhibitors used affected cell viability.

### Determination of MIF and IL-6 Content by Sandwich ELISA

The MIF and IL-6 levels in the conditioned media of the cell cultures were determined by sandwich ELISA using two matched pairs of monoclonal antibodies: anti-human MIF mouse mAb (no. 525501)/anti-human MIF-biotin conjugated mouse mAb (no. 525503) from BioLegend (San Diego) and anti-human IL-6 mouse mAb (no. 21450061)/anti-human IL-6-biotin conjugated mouse mAb (no. 21670062) from ImmunoTools (Friesoythe, Germany) according to the manufacturer’s instructions. The assays were performed in duplicate for each sample of the conditioned medium.

### Determination of PGE_2_ Content by Competitive ELISA

The levels of PGE_2_ in conditioned media were determined by competitive ELISA using the Prostaglandin E_2_ ELISA Kit-Monoclonal (no. 514010) from Cayman Chemical Co. (Ann Arbor, MI), according to the manufacturer’s instructions. The concentration of PGE_2_ was estimated using an inhibition curve, constructed with standard solutions of PGE_2_ (Cayman Chemical Co.). Each assay was performed in duplicate.

### Determination of Gelatinolytic Activity of Matrix Metalloproteinase-2 by Gelatin Zymography

The influence of WISP1 on gelatinolytic activity was determined using the gelatin zymography technique as previously described (50). The enzymatic activity was verified by digital analysis of the intensity of each gelatin lysis band. All samples were run in duplicate.

### Wound Healing Assay

HLFs were cultured in 6-well plates in DMEM/10% FCS until confluent monolayers. After a 16-h starvation period in DMEM/0.1% FCS, to synchronize the cells, a scratch wound assay was performed by creating a wound on the cell monolayers. Subsequently, cells were washed twice with fresh medium to eliminate detached cells and debris, followed by treatment with cytarabine to inhibit cell proliferation. Then, cells were preincubated for 30 min with the Src kinase inhibitor PP2 (1 µM) or the MIF inhibitor ISO-1 (100 μM) in the same medium, followed by stimulation with WISP1 (100 ng/mL) or MIF (100 ng/mL) alone for 0, 24, and 48 h. Cell migration was visualized using an EVOS system microscope, and images were captured at the specified time points to track the closure of the wound over the incubation period. Quantitative analysis of wound closure was performed on the acquired images to assess the impact of WISP1 and MIF stimulation, as well as the inhibitory effects of ISO-1 and PP2 on HLF migration.

### Western Blotting

For Western blotting of MIF, samples of conditioned media containing equal protein concentration, which was determined by the Bradford method (51), were enriched in MIF by precipitation with (NH_4_)_2_SO_4_ (50% saturation). The resulting precipitates were dissolved in Laemmli sample buffer (52), containing 2-mercaproethanol, and then subjected to sodium dodecyl sulfate-polyacrylamide gel electrophoresis (SDS-PAGE) on 15% polyacrylamide gels, followed by Western blotting using biotin-conjugated anti-human MIF mAb (1 μg/mL). For Western blotting of cellular proteins, fibroblasts, upon treatment with WISP1 or MIF, were lysed in 10 mM Tris·HCl (pH 6.8), 2% SDS, 10% glycerol, 100 mM DTT, 2 mM EGTA, and 20 mM NaF. Cell lysates were subjected to SDS-PAGE on 8% polyacrylamide gels followed by Western blot analysis using specific antibodies against NF-κB p65 and NF-κB P-p65, ERK1/2 and P-ERK1/2, Akt and P-Akt, and MIF receptor CD74 and coreceptor CD44 at dilution of 1/1000.

The membranes were incubated overnight at 4°C, followed by incubation with HRP-conjugated streptavidin (immunoblotting of secreted MIF) or HRP-conjugated secondary antibodies (immunoblotting of cellular proteins) at appropriate dilutions for 1 h at room temperature. In the case of MIF receptors, the membranes were also reprobed with an anti-α-tubulin rabbit polyclonal antibody. The immunoreactive protein bands were detected by the enhanced chemiluminescence method, according to the instructions of the manufacturer. The intensity of protein bands was measured using the Scion Image PC software (53) and expressed in arbitrary units (pixels). The ratio of phosphorylated form of the respective transcription factor or kinase-to-total form, as well as of CD74 or CD44/α-tubulin, was also calculated.

### Immunoprecipitation of EGF Receptor and Immunoblotting

Immunoprecipitation (IP) of EGFR was performed as previously described with minor modifications (54). In brief, fibroblasts were cultured in FBS-free DMEM in duplicate with WISP1 in the absence or presence of PP2 or AG-1478 inhibitors for 7 min in 6-well plates. The conditioned medium was removed and the cells were lysed in an ice-cold lysis buffer containing 50 mM Tris·HCl (pH 7.4), 150 mM NaCl, 0.5% NP40, 50 mM NaF, 1 mM Na_3_VO_4_, 1 mM phenylmethylsulfonyl fluoride, 25 μg/mL leupeptin, 5 mM benzamidine-HCl, 10 mM Na_2_EDTA, and 25 μg/mL aprotinin (50 μL/well) (55). The lysates were transferred to microcentrifuge tubes of 1.5 mL, vortexed, and clarified by centrifugation at 14,000 *g* for 30 min at 4°C. IP was performed by adding 1 μL (1 μg) of anti-EGFR rabbit polyclonal antibody (IP specific) and 30 μL of protein A agarose bead slurry to each 90 μL cell lysate sample on ice bath and incubating at 4°C with gentle rocking overnight. Samples were then centrifuged at 14,000 *g* for 2 min at 4°C. The pellets were washed three times with 200 μL each time of ice-cold cell lysis buffer, resuspended in 30 μL of 2× Laemmli samples buffer, vortexed, boiled for 5 min, and centrifuged for 2 min at 14,000 *g*, and the supernatants were collected and subjected to SDS-PAGE and Western blotting as described above, using mouse mAb against P-Tyr and rabbit mAb against EGFR.

### Quantitative Real-Time PCR

Total RNA was extracted from fibroblasts using a Nucleo Spin RNA kit (Macherey-Nagel, Düren, Germany), according to the instructions of the manufacturer, and was used for cDNA synthesis by the Prime Script RT Reagent Kit (Takara, Nippon, Japan), according to the protocol of the manufacturer. The quantitative real-time PCR (qPCR) analysis was performed, using the KAPA SYBR FAST qPCR Master Mix (2×) kit (KAPA Biosystems, Boston, MA), according to the instructions of the manufacturer. Assays were carried out in triplicate on a Rotor-Gene Q detection system (Qiagen) in a total volume of 20 μL, including 10 μL KAPA SYBR FAST qPCR Master Mix (2×), 0.5 μL of each primer of concentration 8 μM, 1–2 μL cDNA (0.5–20 ng), and 7 or 8 μL of dH_2_O. The cycling conditions were 3-min enzyme activation at 95°C, followed by 40 cycles at 95°C for 3 s and 50–60°C for 20 s. GAPDH was used as an internal standard. The primer sequences were as follows: MIF, forward: 5′-CCG GAC AGG GTC TAC ATC AAC TAT TAC-3′ and reverse: 5′-TAG GCG AAG GTG GAG TTG TTC C-3′; CD74, forward: 5′-TGC ATT CAC ATT TGT GCT GTA G-3′ and reverse: 5′-TGT ACA GAG CTC TCC ACG GCT G-3′; CD44 (total), forward: 5′-ATA ATT GCC GCT TTG CAG GTG TAT T ′ and reverse: 5′-ATA ATG GCA AGG TGC TAT TGA AAG CCT-3′; Fyn, forward: 5′-TGT GAC CTC CAT CCC CAA CT-3′ and reverse: 5′-AAC TCA GGT CAT CTT CTG TCC GT-3′; Lyn, forward: 5′-GAG GCT CTA CGC TGT GGT CA-3′ and reverse: 5′-GAC TCG GAG ACC AGA ACA TTA GC-3′; IL-6, forward: 5′-TCC AGA ACA GAT TTG AGA GTA GTG-3′ and reverse: 5′-GCA TTT GTG GTT GGG TCA GG-3′; COX-2, forward: 5′-GCC AGT GAA TCC CTG TTG TTA CT-3′ and reverse: 5′-GGC CGA AGC GGA CAC A-3′; matrix metalloproteinase (MMP)-2, forward: 5′-ACT GTT GGT GGG AAC TCA GAA G-3′ and reverse: 5′-CAA GGT CAA TGT CAG GAG AGG-3′; and GAPDH, forward: 5′-AGG CTG TTG TCA TAC TTC TCA T-3′ and reverse: 5′-GGA GTC CAC TGG CGT CTT-3′. Quantification of gene expression levels was analyzed using the ΔΔCt method developed by Livak and Schmittgen (56).

### Statistical Analysis

Data were analyzed using the unpaired Student’s *t* test, with a limit of significance at *P* < 0.05.

## RESULTS

### Dose- and Time-Dependent Effect of WISP1 on the Expression of MIF

**Figure 1.**
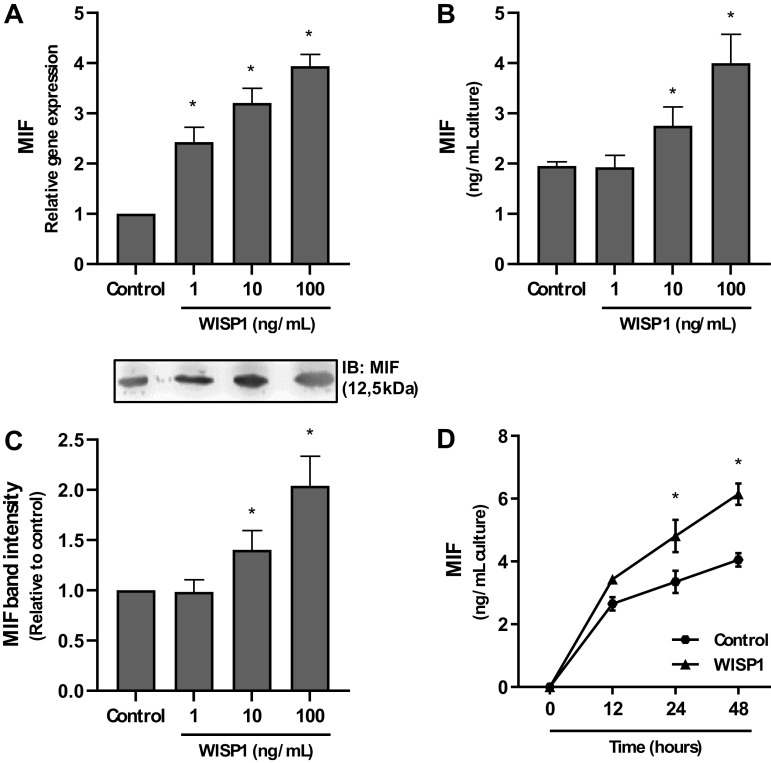
Concentration- and time-dependent effects of Wnt1-inducible signaling protein 1 (WISP1) on the expression of migration inhibitory factor (MIF) in human lung fibroblasts (HLFs). *A*–*C*: HLFs were cultured with different concentrations (0-100 ng/mL) of WISP1 for 48 h, and the expression of MIF at both the mRNA and protein levels was examined by quantitative PCR and ELISA/immunoblotting (IB), respectively. *D*: HLFs were cultured with WISP1 (100 ng/mL) for different time periods (0, 12, 24, and 48 h), and the secreted levels of MIF were determined by ELISA. Data represent the means ± SD (*n* = 3). *Statistically significant increase (*P* < 0.05) compared to control.

HLFs were cultured in the presence of WISP1 (1 to 100 ng/mL) for 48 h or WISP1 (100 ng/mL) for different time periods (0–48 h), and the expression of MIF was examined (Fig. 1). We found that WISP1 significantly induced the production of MIF in a concentration- and time-dependent manner.

### Involvement of αvβ5-Integrin and Chondroitin Sulfate Proteoglycans in WISP1-Induced Expression of MIF

Previous studies have shown that the CCN family of proteins affects cellular functions by binding to cell surface integrins. In particular, CCN1 showed proangiogenic activities in HUVEC cells through binding to αvβ3- and α6β1-integrins (57), while WISP1 (CCN4) induced IL-6 expression in fibroblasts from synovial membrane from patients with osteoarthritis (OASMFs) through αvβ5-integrin (58). It has also been reported that WISP1 binds to decorin and biglycan proteoglycans secreted in the conditioned media of human skin fibroblasts thus modulating its interactions with the surface of fibroblasts (59).

**Figure 2.**
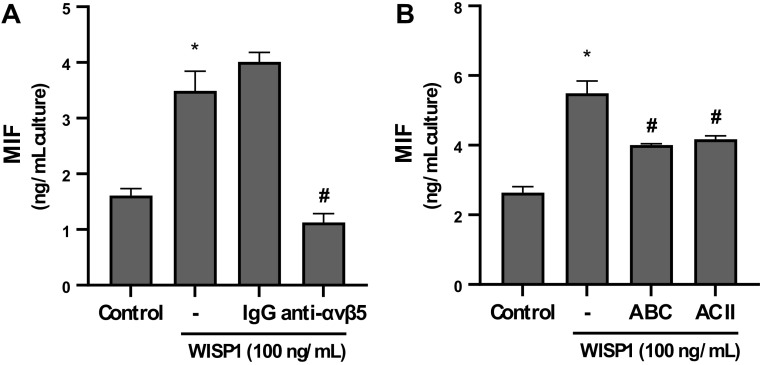
αvβ5-integrin and chondroitin sulfate proteoglycans are implicated in Wnt1-inducible signaling protein 1 (WISP1)-induced production of migration inhibitory factor (MIF) from human lung fibroblasts (HLFs). HLFs were pretreated for 30 minutes with anti-αvβ5-integrin antibody (6 µg/mL) (*A*) or for 2 h with chondroitinase ABC or chondroitinase AC II (3 units/mL) (*B*), followed by stimulation with WISP1 (100 ng/mL) for 48 h, and the levels of MIF in conditioned media were determined by ELISA. Data represent the means ± SD (*n* = 3). *Statistically significant increase (*P* < 0.05) compared to control. #Statistically significant decrease (*P* < 0.05) compared to WISP1-treated cells. IgG represents immunoglobulins from nonimmunized mouse.

Considering the above data, HLFs were pretreated with a mAb against αvβ5-integrin or with chondroitinase ABC (degrades chondroitin/dermatan sulfate side chains) or chondroitinase AC II (degrades exclusively chondroitin sulfate side chains) followed by stimulation with WISP1, and MIF levels in the conditioned media were determined by ELISA (Fig. 2, *A* and *B*, respectively). All treatments significantly inhibited WISP1-induced expression of MIF. Since the IgG from nonimmunized mouse did not affect the stimulatory effect of WISP1 on the production of MIF, as well as the two chondroitinases had the same suppressive effect on the expression of MIF, these results suggest that WISP1-induced expression of MIF is mediated by αvβ5-integrin and chondroitin sulfate proteoglycans.

### Protein Kinases Are Implicated in WISP1-Induced Expression of MIF

**Figure 3.**
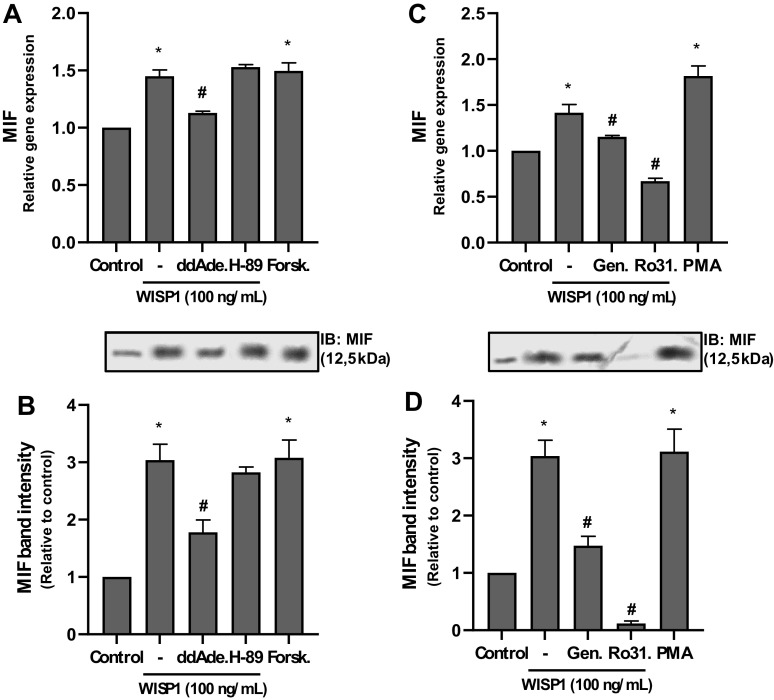
cAMP pathway, PKC, and tyrosine kinases are implicated in Wnt1-inducible signaling protein 1 (WISP1)-induced expression of migration inhibitory factor (MIF) in human lung fibroblasts (HLFs). *A* and *B*: HLFs were pretreated for 30 minutes with adenylate cyclase inhibitor 2′,5′-dideoxy-adenosine (ddAde.; 10 µM) or PKA inhibitor H-89 (5 µM) followed by stimulation with WISP1 (100 ng/mL) for 48 or were stimulated with adenylate cyclase activator forskolin (Forsk.; 10 µM) alone for 48 h and the expression of MIF at both the mRNA and protein levels was examined by quantitative PCR (qPCR) and immunoblotting (IB), respectively. *C* and *D*: HLFs were pretreated for 30 minutes with the PKC inhibitor Ro31-8220 (Ro31.; 5 µM) or the tyrosine kinase inhibitor genistein (Gen.; 100 µM) followed by stimulation with WISP1 (100 ng/mL) for 48 h or were stimulated with PKC activator PMA (100 nM) alone for 48 h, and the expression of MIF at both the mRNA and protein levels was examined by qPCR and IB, respectively. Data represent the means ± SD (*n* = 3). *Statistically significant increase (*P* < 0.05) compared to control. #Statistically significant decrease (*P* < 0.05) compared to WISP1-treated cells.

It has been reported that several response elements, such as AP1, AP4, CREB, NF-κB, and STAT, are located within the promoter of MIF (60, 61). In addition, PKC has been reported to be involved in the expression of MIF in various cells, such as eosinophils (44), and in neural (62) and cardiac muscle cells (63); in the latter, the involvement of tyrosine kinases, in particular Src kinases, has also been reported (64). Considering these data, we next investigated the implication of PKA, PKC, and tyrosine kinases in WISP1-induced expression of MIF. HLFs were pretreated with an inhibitor of PKA (H-89) or PKC (Ro31-8220) or tyrosine kinases (genistein), followed by stimulation with WISP1, and the expression of MIF was examined (Fig. 3). As shown in Fig. 3, *A* and *B*, H-89 had no effect on the expression of MIF, suggesting that PKA is not involved. However, when fibroblasts were cultured only in the presence of forskolin, an adenylate cyclase activator, it was observed that the expression of MIF was stimulated at levels similar to those induced by WISP1, suggesting the involvement of cAMP in the expression of MIF (Fig. 3, *A* and *B*). This suggestion was further supported by pretreatment of cells with the adenylate cyclase inhibitor, 2′,5′-dideoxy adenosine. As shown in Fig. 3, *A* and *B*, this inhibitor caused a partial but statistically significant suppression of MIF expression, indicating that cAMP is indeed involved in WISP1-induced expression of MIF. However, since the cAMP/PKA pathway is not involved, as mentioned above, it is possible that another pathway activated by cAMP, such as the cAMP/EPAC pathway, might be implicated in WISP1-induced expression of MIF, but this remains to be elucidated.

As shown in Fig. 3, *C* and *D*, both the PKC inhibitor (Ro31-8220) and the tyrosine kinase inhibitor (genistein) caused significant suppression of MIF expression, suggesting that both PKC and tyrosine kinases are involved in WISP1-induced expression of MIF. Moreover, the observation that Ro31-8220 strongly inhibited the expression of MIF below the basal level indicates probably that PKC is involved in the constitutive expression of MIF in HLFs. However, this requires further investigation. The implication of PKC was further supported using the PKC activator PMA. When cells were cultured with PMA alone a significant stimulation of MIF expression was observed at levels similar to those induced by WISP1 (Fig. 3, *C* and *D*).

### Src Kinases Are Implicated in WISP1-Induced Expression of MIF

As shown previously, in WISP1-induced expression of MIF in HLFs, αvβ5-integrin as well as tyrosine kinases were implicated. It is known that αvβ5-integrin signaling is mediated by Src tyrosine kinases in various cells, such as endothelial and breast cancer cells (65, 66), and specific Src kinase family members are involved in the expression of MIF in cardiac muscle cells (64). Based on the above data, it was investigated whether Src kinases are involved in WISP1-induced expression of MIF in HLFs.

**Figure 4.**
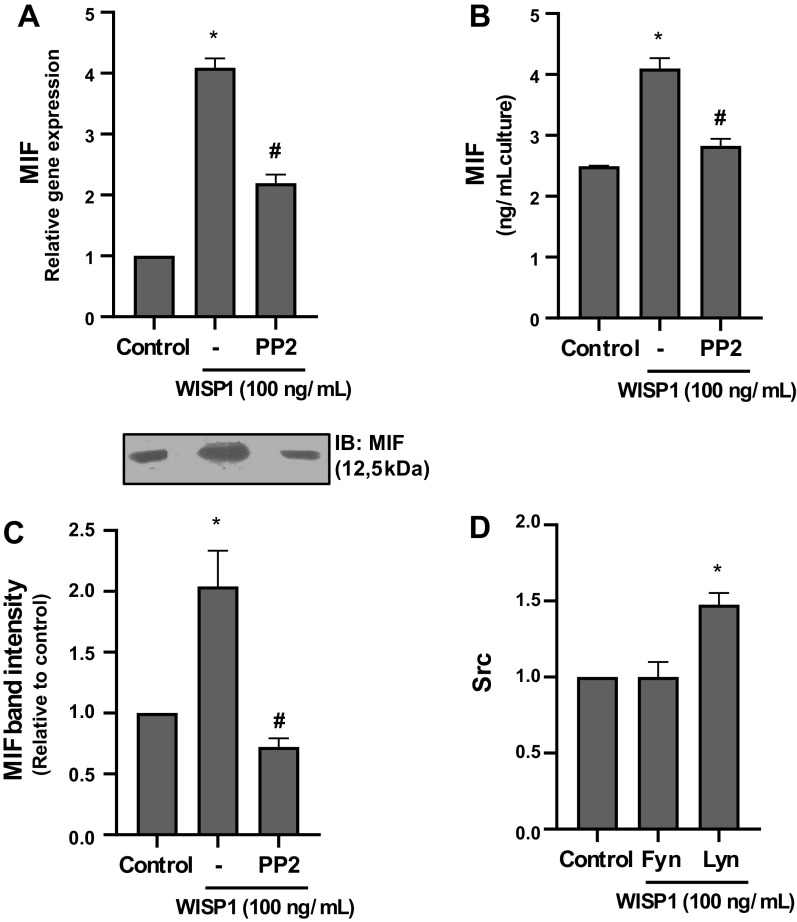
Src kinases are implicated in Wnt1-inducible signaling protein 1 (WISP1)-induced expression of migration inhibitory factor (MIF) in human lung fibroblasts (HLFs). *A*–*C*: HLFs were pretreated for 30 minutes with the Src kinase inhibitor PP2 (1 µM) followed by stimulation with WISP1 (100 ng/mL) for 48 h, and the expression of MIF at both the mRNA and protein levels was examined by quantitative PCR (qPCR) and ELISA/immunoblotting (IB), respectively. *D*: HLFs were treated with WISP-1 (100 ng/mL) for 48 h, and the mRNA expression of Src kinases Fyn and Lyn was examined by qPCR analysis. Data represent the means ± SD (*n* = 3). *Statistically significant increase (*P* < 0.05) compared to control. #Statistically significant decrease (*P* < 0.05) compared to WISP1-treated cells.

HLFs were pretreated with the selective inhibitor of Src kinase PP2 followed by stimulation with WISP1, and the expression of MIF was examined (Fig. 4, *A*–*C*). The results showed that PP2 caused almost complete suppression of MIF expression, suggesting that Src kinases are indeed involved and play a pivotal role in WISP1-induced expression of MIF in HLFs.

The involvement of Src kinases in WISP1-induced expression of MIF was further supported by studying the effect of WISP1 on the expression of the Src kinases Fyn and Lyn in HLFs by qPCR analysis (Fig. 4*D*). It was observed that WISP1 caused a significant enhancement in the expression of Lyn, whereas it had no effect on Fyn expression. Consequently, it could be suggested that Lyn kinase is possibly among the Src kinases involved in WISP1-induced expression of MIF. However, this suggestion should be further explored and is currently under investigation.

### Involvement of NF-κB in WISP1-Induced Expression of MIF

WISP1, acting through different cell surface integrins, exhibits diverse actions. From previous studies, it is known that in various pathological conditions, and depending on the cell type, it can activate various signaling pathways, among them the NF-κB pathway (67). As mentioned previously, the NF-κB response element is found in the promoter of MIF (60, 61). In addition, NF-κB was shown to be involved in WISP1-induced expression of IL-6 in OASMFs (58).

**Figure 5.**
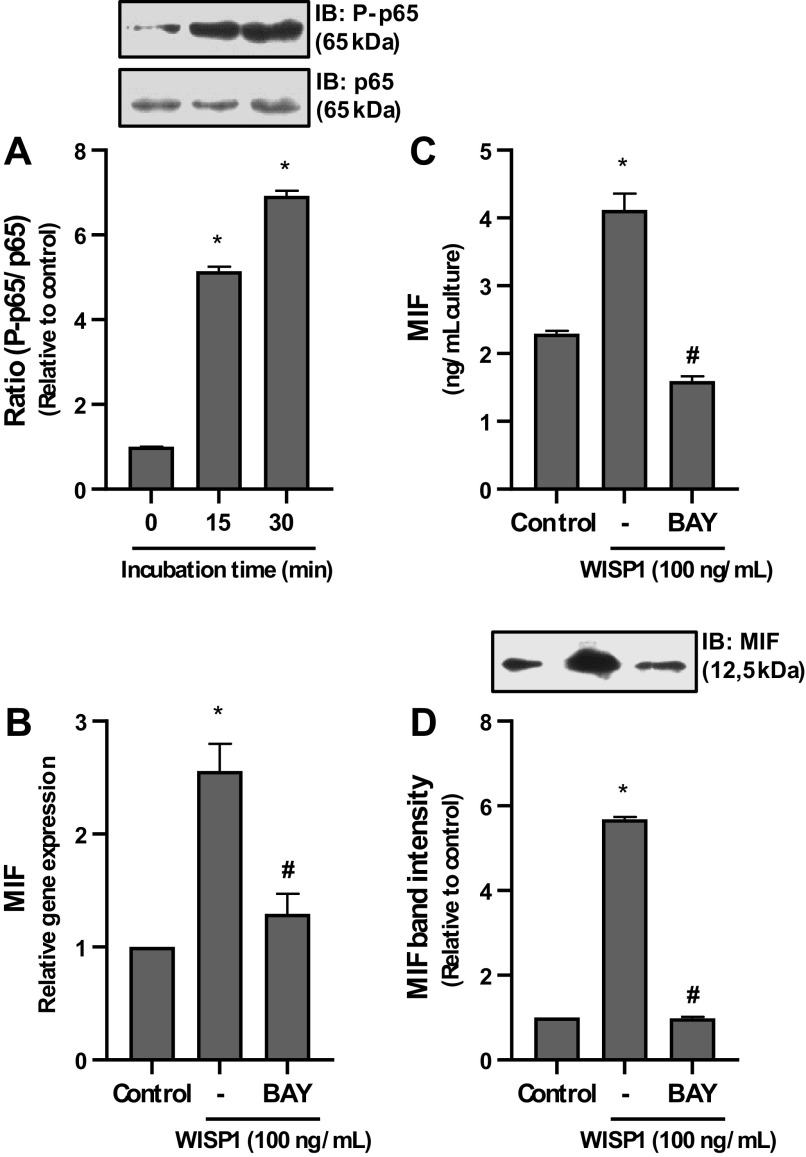
NF-κB is implicated in Wnt1-inducible signaling protein 1 (WISP1)-induced expression of migration inhibitory factor (MIF) in human lung fibroblasts (HLFs). *A*: HLFs were treated with WISP1 for the indicated time intervals and P-p65 was determined by immunoblotting (IB). *B*–*D*: HLFs were pretreated for 30 minutes with the NF-κB activation inhibitor Bay-11-7082 (BAY; 5 µM) followed by stimulation with WISP1 (100 ng/mL) for 48 h, and the expression of MIF at both the mRNA and protein levels was examined by quantitative PCR and ELISA/IB, respectively. Data represent the means ± SD (*n* = 3). *Statistically significant increase (*P* < 0.05) compared to time zero (*A*) or control (*B* and *C*). #Statistically significant decrease (*P* < 0.05) compared to WISP1-treated cells.

In view of these data, we investigated whether WISP1 can activate the transcription factor NF-κB and whether NF-κB is involved in WISP1-induced expression of MIF in HLFs. Cells were cultured with WISP1 for different time periods, lysed, and extracted, and the levels of phosphorylated p65 (P-p65) regulatory subunit of NF-κB, relative to total p65, were determined by immunoblotting (Fig. 5*A*). The P-p65/total p65 ratio revealed that WISP1 caused a statistically significant time-dependent increase in the levels of P-p65 in fibroblasts, which peaked after 30 min in culture, suggesting the ability of WISP1 to activate the NF-κB pathway. Subsequently, the involvement of WISP1-activated NF-κB in WISP1-induced expression of MIF was investigated. HLFs were pretreated with the NF-κB activation inhibitor Bay-11-7082, followed by stimulation with WISP1, and the expression of MIF was examined (Fig. 5, *B*–*D*). The inhibitor caused almost complete suppression of MIF expression suggesting the pivotal role of NF-κB in WISP1-induced expression of MIF in HLFs.

### WISP1 Activates PKB/Akt and ERK1/2 Pathways

It has been previously reported that WISP1 regulates the growth and survival of several cells through the activation of a variety of signaling pathways, including the PI3K/Akt pathway (7, 10–12, 68). The PI3K/Akt pathway is also known to be involved in the WISP1-induced expression of IL-6 in OASMFs (58). Moreover, it is known that WISP1 can activate the MEK/ERK pathway in human glioblastoma and lung epithelial cells (14, 69).

**Figure 6.**
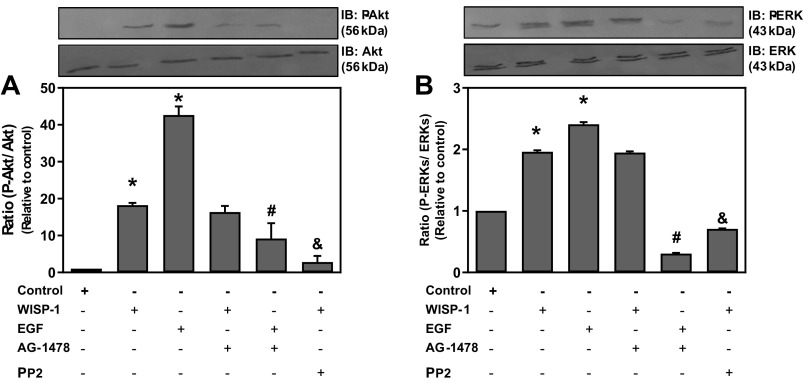
Wnt1-inducible signaling protein 1 (WISP1) activates ERK1/2 and Akt. Human lung fibroblasts (HLFs) were pretreated for 30 minutes with the Src kinase inhibitor PP2 (1 µM) or with the EGF receptor tyrosine kinase activity inhibitor AG-1478 (1 µM) followed by stimulation with WISP1 or EGF (100 ng/mL) each for 7 minutes, and P-Akt (*A*) and P-ERK1/2 (*B*) were determined by immunoblotting (IB). Data represent the means ± SD (*n* = 3). *Statistically significant increase (*P* < 0.05) compared to control. #Statistically significant decrease (*P* < 0.05) compared to EGF-treated cells. &Statistically significant decrease (*P* < 0.05) compared to WISP1-treated cells.

To investigate whether the PI3K/Akt and MEK/ERK pathways are implicated in WISP1-induced expression of MIF, HLFs were pretreated with the Src inhibitor PP2 or the specific EGFR tyrosine kinase activity inhibitor AG-1478, followed by stimulation with WISP1 or EGF. Upon lysis and extraction of cells, the cell extracts were subjected to Western blotting using antibodies against phosphorylated Akt (P-Akt) and total Akt, or antibodies against phosphorylated ERK1/2 (P-ERK1/2) and total ERK1/2 (Fig. 6). The stimulation with EGF was used as positive control since it is known that EGF activates both PI3K/Akt and MEK/ERK pathways.

From the intensity ratio of P-Akt protein band to that of total Akt, or P-ERK1/2 to total ERK1/2, a statistically significant increase in P-Akt and P-ERK1/2 was observed upon stimulation with either WISP1 or EGF. The EGF-induced phosphorylation of both Akt and ERK1/2 was completely abolished in the presence of AG-1478 inhibitor, as expected. The WISP1-induced phosphorylation of Akt or ERK1/2 was strongly suppressed by the PP2 inhibitor, while the AG-1478 inhibitor had no statistically significant effect. These results indicate that WISP1 can induce Akt and ERK1/2 phosphorylation probably through activation of EGFR regardless of the presence of a EGFR ligand.

### MAP Kinases and PI3K Are Implicated in WISP1-Induced Expression of MIF

**Figure 7.**
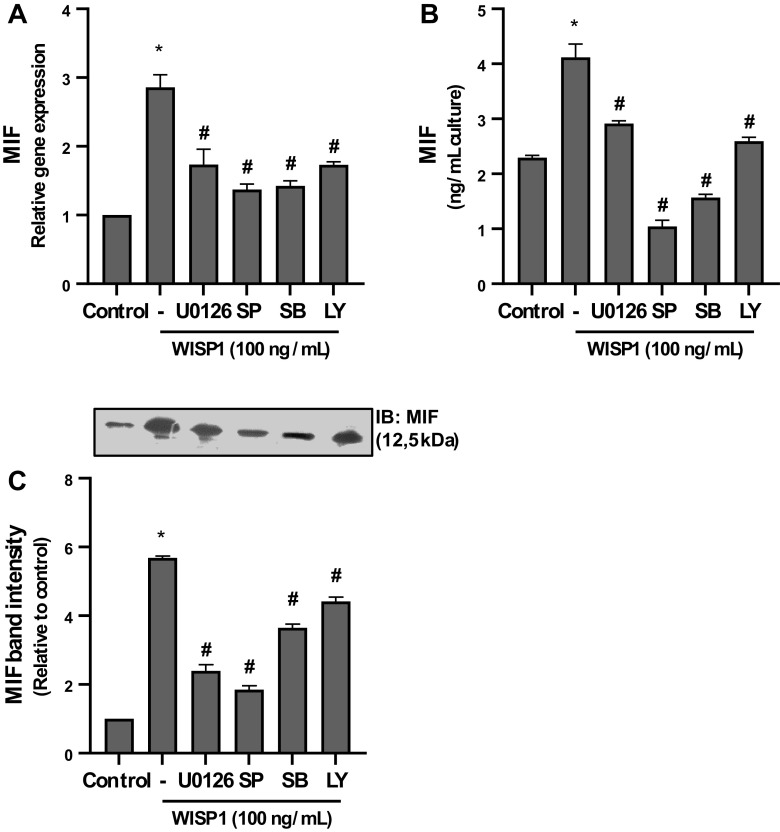
MAP kinases (MAPKs) and phosphatidylinositol 3-kinase (PI3K) are implicated in Wnt1-inducible signaling protein 1 (WISP1)-induced expression of migration inhibitory factor (MIF) in human lung fibroblasts (HLFs). *A–C*: HLFs were pretreated for 30 minutes with the MAPK inhibitors U0126 (10 μM), SP600125 (SP; 20 μM), SB203580 (SB; 10 μM), and the PI3K inhibitor LY294002 (LY; 10 μΜ) followed by stimulation with WISP1 (100 ng/mL) for 48 h and the expression of MIF at both the mRNA and protein levels was examined by quantitative PCR and ELISA/immunoblotting (IB), respectively. Data represent the means ± SD (*n* = 3). *Statistically significant increase (*P* < 0.05) compared to control. #Statistically significant decrease (*P* < 0.05) compared to WISP1-treated cells.

Given the ability of WISP1 to activate Akt and ERK1/2, we next investigated the possible role of PI3K/Akt and ERK1/2 as well as of other MAP kinases in the upregulation of MIF by WISP1, using specific kinase inhibitors. To this aim, HLF were pretreated with LY294002, U0126, SP600125, and SB203580, inhibitors of PI3K, MEK1/2, JNK, and p38 kinases, respectively, followed by stimulation with WISP1 and the expression of MIF was examined. As shown in Fig. 7, *A*–*C*, all tested inhibitors caused statistically significant suppression of WISP1-induced expression of MIF, indicating that except for PI3K/Akt and MEK/ERK1/2 signaling pathways, the JNK and p38 kinases may be also implicated in the expression of MIF.

### EGF Can Stimulate MIF Expression in HLF

**Figure 8.**
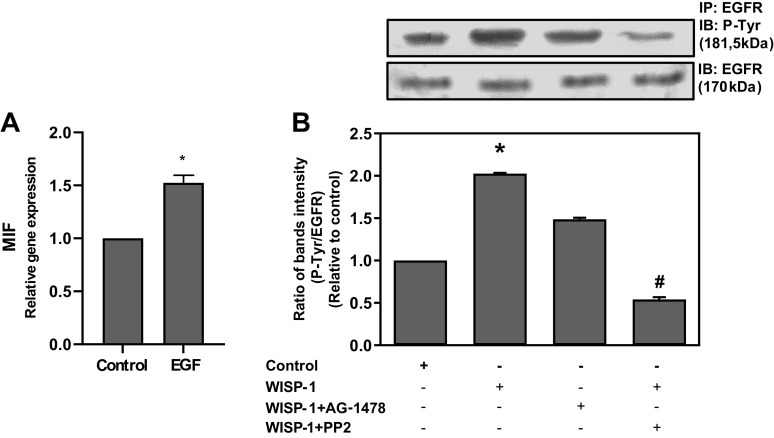
EGF is able to induce the expression of migration inhibitory factor (MIF), and EGF receptor (EGFR) is activated by WISP1 through transactivation by Src kinases in human lung fibroblasts (HLFs). *A*: HLFs were stimulated with EGF (100 ng/mL) for 48 h, and the mRNA expression of MIF was examined by quantitative PCR. Data represent the means ± SD (*n* = 3). *Statistically significant increase (*P* < 0.05) compared to control. *B*: HLFs were pretreated for 30 minutes with the Src kinase inhibitor PP2 (1 µM) or with the EGFR tyrosine kinase activity inhibitor AG-1478 (1 µM) followed by stimulation with WISP1 (100 ng/mL) for 5 minutes. Then, cells were lysed and extracted, and the cell extracts were subjected to immunoprecipitation, using a polyclonal antibody against EGFR and a protein A agarose bead slurry. After centrifugation, the pellets were treated with Laemmli sample buffer and subjected to immunoblotting (IB) using mouse mAb against phosphotyrosine (P-Tyr) and rabbit mAb against EGFR. Data represent the means ± SD (*n* = 3) of the P-EGFR/total EGFR ratio. *Statistically significant increase (*P* < 0.05) compared to control. #Statistically significant decrease (*P* < 0.05) compared to WISP1-treated cells.

It has been previously reported that EGF is able to induce MIF expression in MCF10CAT breast cancer cells as well as in A431 cervical cancer cells through EGFR/MEK/ERK1/2 signaling pathway (70). Given that EGF induces the activation of Akt and ERK1/2 pathways, which are involved in MIF expression in HLFs, as shown in Fig. 7, it was investigated whether EGF has the ability to induce MIF expression in HLFs as well. Fibroblasts were cultured with EGF, and MIF expression at the mRNA level was ascertained by qPCR analysis (Fig. 8*A*). It was observed that EGF caused a significant increase (∼50%) in the expression of MIF, revealing that EGF indeed is able to stimulate the expression of MIF in HLFs.

### WISP1 Activates EGFR through Transactivation by Src Kinases

The above results indicate that the WISP1-induced expression of MIF in HLFs might be mediated by EGFR activation. It has been reported that Src kinases, regardless of the presence of an EGFR ligand, can activate (transactivate) this receptor (71, 72) and that in lung adenocarcinoma cells the Src kinase responsible for the activation of EGFR is Lyn (73). Given that WISP1 induces the expression and activation of Src kinases in HLFs (Fig. 4), it was investigated whether WISP1 is able to activate EGFR through transactivation by Src kinases. HLFs were cultured with WISP1 in the absence and presence of PP2 or AG-1478, followed by cell lysis and extraction, immunoprecipitation of EGFR, as well as immunoblotting of phosphorylated tyrosines (P-Tyr), and EGFR was performed, as described in materials and methods. The ratio of P-Tyr on EGFR to total EGFR revealed that WISP1 caused a significant increase in EGFR phosphorylation, which was completely suppressed in the presence of the Src kinase inhibitor PP2, and to a lesser extent by the EGFR inhibitor AG-1478 (Fig. 8*B*). These results demonstrate that WISP1 activates EGFR through transactivation by Src kinases resulting in the subsequent induction of MIF expression in HLFs via EGFR-activated pathways in a ligand-independent manner.

### MIF Is Able to Induce Its Own Expression

**Figure 9.**
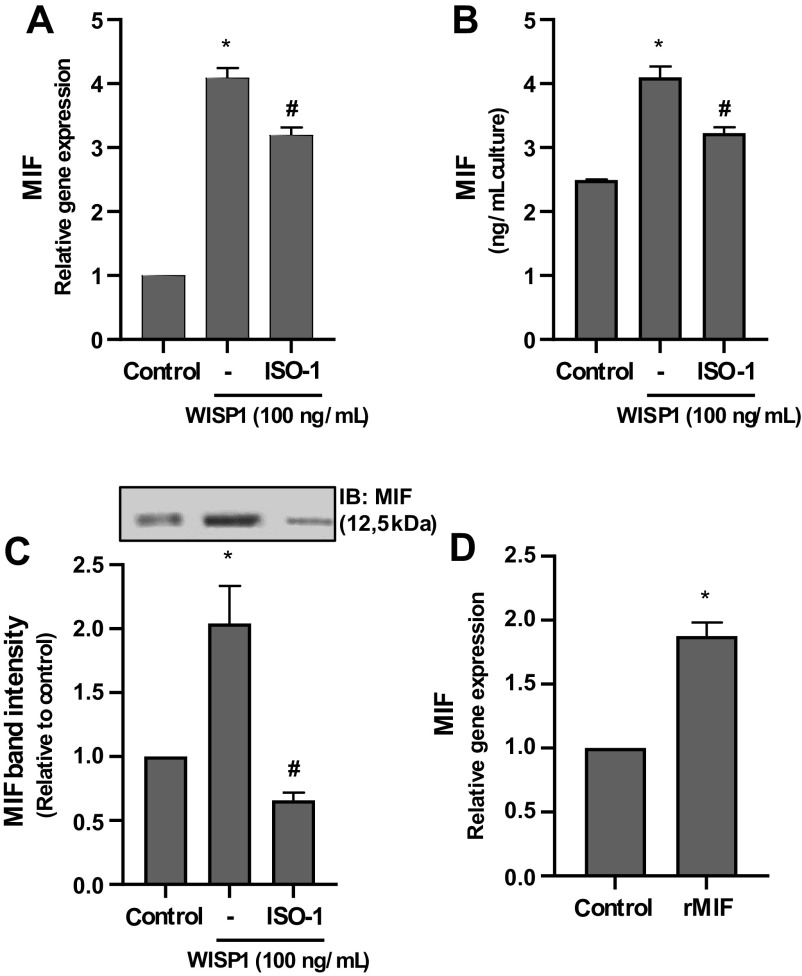
Migration inhibitory factor (MIF) is able to stimulate its own gene expression in human lung fibroblasts (HLFs). *A*–*C*: HLFs were pretreated for 30 minutes with the MIF inhibitor ISO-1 (100 μM), followed by stimulation with WISP1 (100 ng/mL) for 48 h, and the expression of MIF at both the mRNA and protein levels was examined by quantitative PCR (qPCR) and ELISA/immunoblotting (IB), respectively. Data represent the means ± SD (*n* = 3). *Statistically significant increase (*P* < 0.05) compared to control. #Statistically significant decrease (*P* < 0.05) compared to WISP1-treated cells. *D*: HLFs were stimulated with recombinant MIF (rMIF) protein (100 ng/mL) for 48 h, and the mRNA expression of MIF was examined by qPCR. Data represent the means ± SD (*n* = 3). *Statistically significant increase (*P* < 0.05) compared to control.

It has been reported that MIF acts through CD74 receptor, which is found in complexes with the hyaluronan receptor CD44. After binding of MIF to its receptor, the cytoplasmic domain of CD44 interacts with tyrosine kinases of the Src family and causes changes in their conformation, resulting in the disclosure of their active site and their activation by autophosphorylation. Activated Src kinases can then activate various signaling pathways (74–76). According to our data, Src kinases are strongly involved in the WISP1-induced expression of MIF. Therefore, it was supposed that the produced MIF might induce its own expression, acting in an autocrine manner through its receptors. This hypothesis is further enhanced by previously reported data showing that recombinant MIF in cardiac fibroblast cultures could induce its own expression (77). To investigate this hypothesis, HLFs were pretreated with the specific inhibitor of MIF ISO-1 followed by stimulation with WISP1, and the expression of MIF was examined (Fig. 9). It was found that the ISO-1 inhibitor caused a significant suppression of WISP1-induced expression of MIF, suggesting that the produced MIF is implicated in its own expression (Fig. 9, *A*–*C*). This was further investigated by stimulation of HLF with recombinant MIF (rMIF) alone. As shown in Fig. 9*D*, the exogenously added rMIF caused a statistically significant enhancement of the expression MIF, indicating that MIF indeed can induce its own expression in HLFs.

### WISP1 Can Induce the Expression of MIF Receptors

**Figure 10.**
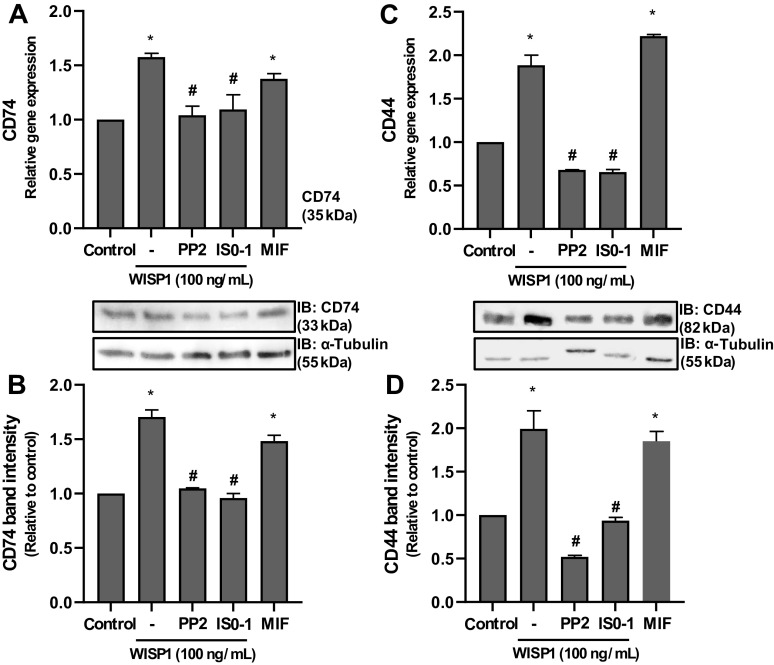
Wnt1-inducible signaling protein 1 (WISP1) and migration inhibitory factor (MIF) induce the expression of CD74 and CD44 receptors in human lung fibroblasts (HLFs). HLFs were pretreated for 30 minutes with the Src kinase inhibitor PP2 (1 µM) or the MIF inhibitor ISO-1 (100 μM), followed by stimulation with WISP1 (100 ng/mL) for 48 h, or cells were stimulated with rMIF (100 ng/mL) alone for 48 h, and the expression of CD74 or CD44 at both the mRNA and protein levels was examined by quantitative PCR (*A* and *B*) and immunoblotting (IB; *C* and *D*), respectively. Data represent the means ± SD (*n* = 3). *Statistically significant increase (*P* < 0.05) compared to control. #Statistically significant decrease (*P* < 0.05) compared to WISP1-treated cells.

Next, the effect of WISP1 on the expression of MIF receptor (CD74) and coreceptor (CD44) was investigated. HLFs were cultured with WISP1, in the absence and presence of PP2 or ISO-1 inhibitors, and the expression of the CD74 and CD44 receptors was examined at both the mRNA and protein levels by qPCR and Western blotting analyses (Fig. 10, *A*–*D*). WISP1 caused a statistically significant induction of the expression of both CD74 and CD44, which was almost completely abolished in the presence of both inhibitors. These results suggest that WISP1 induces the expression of CD74 and CD44 through pathways involving Src kinases and MIF. The latter was further established by culturing HLFs with MIF alone. Cells were cultured with rMIF and the expression of receptors was examined at both the mRNA (Fig. 10, *A* and *B*) and protein levels (Fig. 10, *C* and *D*) by qPCR and Western blotting, respectively. Like WISP1, MIF caused a statistically significant induction of the expression of both receptors, suggesting that the WISP1-induced expression of CD74/CD44 receptors may be mediated by MIF.

### WISP1 Induces the Expression of COX-2, PGE_2_, IL-6, and MMP-2 through MIF

It has been previously reported that prostaglandin E2 (PGE_2_), one of the main mediators of inflammation, can activate Src kinases through EP3 receptors, which in turn transactivate EGFR (regardless of the presence of a ligand) in lung adenocarcinoma cells (78, 79). In addition, several studies have reported that WISP1 enhances IL-6 production by synovial and lung fibroblasts (13, 58). Similarly, MIF has been shown to induce the expression of IL-6 in various cells (80–83).

Matrix metalloproteinases (MMPs), such as MMP-2, play a prominent role in the remodeling of the ECM, which is a main characteristic of various lung diseases. Notably, it has been shown that overexpression of WISP1 in mouse knee joints leads to cartilage degradation by inducing the expression of MMPs (84).

**Figure 11.**
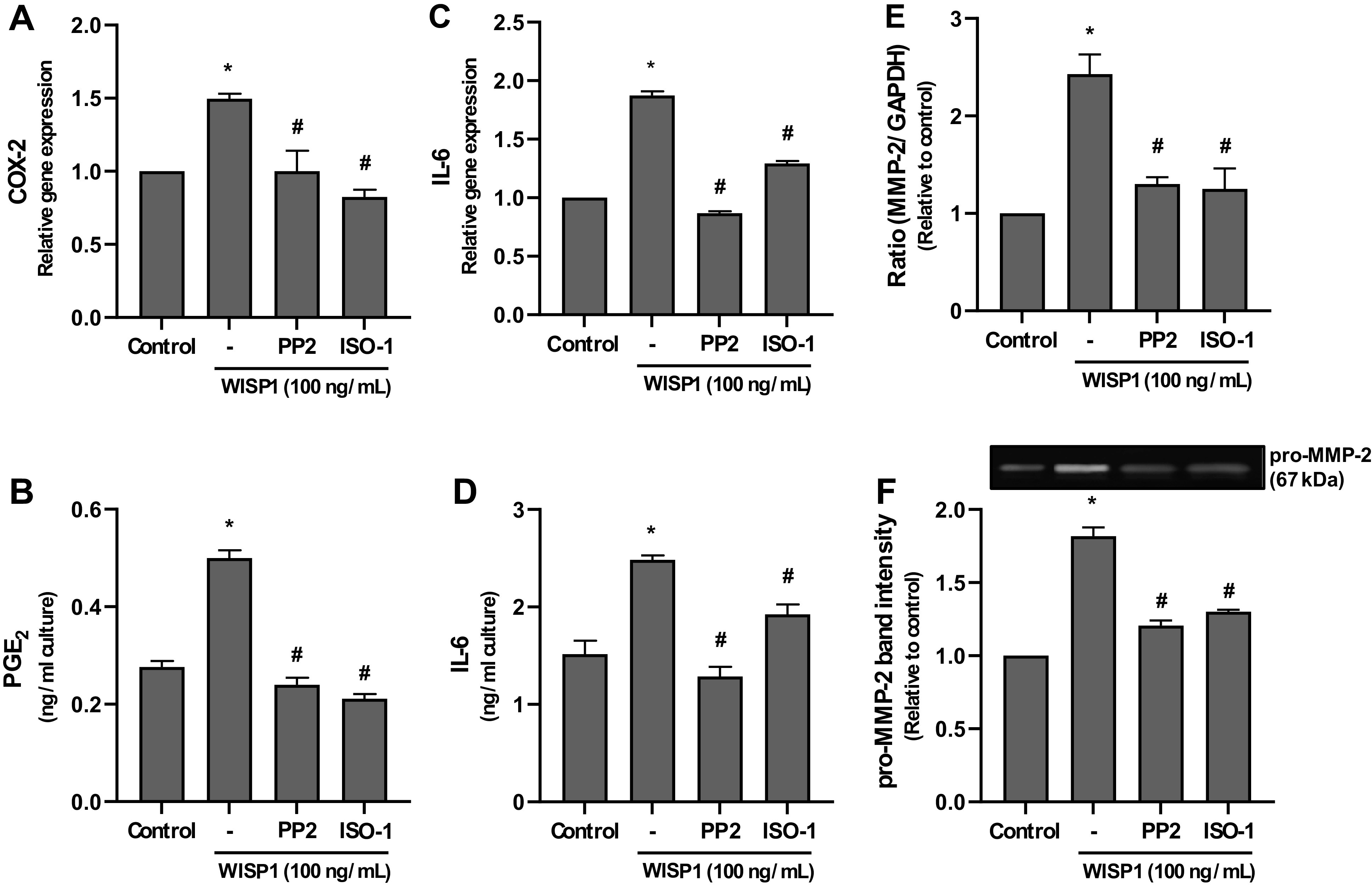
Wnt1-inducible signaling protein 1 (WISP1) induces the expression of cyclooxygenase (COX)-2, IL-6, and matrix metalloproteinase (MMP)-2 and the production of prostaglandin E_2_ (PGE_2_) from HLFs. Human lung fibroblasts (HLFs) were pretreated for 30 minutes with the Src kinase inhibitor PP2 (1 µM) or the MIF inhibitor ISO-1 (100 µM) followed by stimulation with WISP1 (100 ng/mL) for 48 h; the mRNA expression of COX-2, IL-6, and MMP-2 was examined by quantitative PCR (*A*, *C*, and *E*); and the levels of PGE_2_, IL-6, and pro-MMP-2 in conditioned media were determined by ELISA (*B* and *D*) and gelatin zymography (*F*), respectively. Data represent the means ± SD (*n* = 3). *Statistically significant increase (*P* < 0.05) compared to control. #Statistically significant decrease (*P* < 0.05) compared to WISP1-treated cells.

Based on these observations, we examined the ability of WISP1 to induce COX-2, IL-6, and MMP-2 expression, and PGE2 production from HLFs and whether MIF is involved in these effects. HLFs were cultured with WISP1, in the absence and presence of PP2 or ISO-1 inhibitors, and the mRNA expression of COX-2, IL-6, and MMP-2 was examined by qPCR analysis, and the levels of PGE_2_, IL-6, and pro-MMP-2 in conditioned media were determined by ELISA and gelatin zymography, respectively. As shown in Fig. 11, at the mRNA level, WISP1 caused a statistically significant induction of the expression of COX-2, IL-6, and MMP-2, which was almost completely suppressed in the presence of both inhibitors (Fig. 11, *A*, *C*, and *E*). Identically, at the protein level, WISP1 enhanced the production of PGE_2_, IL-6, and pro-MMP-2 from fibroblasts, which was also almost completely suppressed in the presence of the inhibitors (Fig. 11, *B*, *D*, and *F*). Taken together, these findings indicate that WISP1 can trigger the expression of COX-2, PGE2, IL-6, and MMP-2 through the activities of Src kinases and MIF.

### WISP1-Induced MIF Promotes Lung Fibroblast Migration

**Figure 12.**
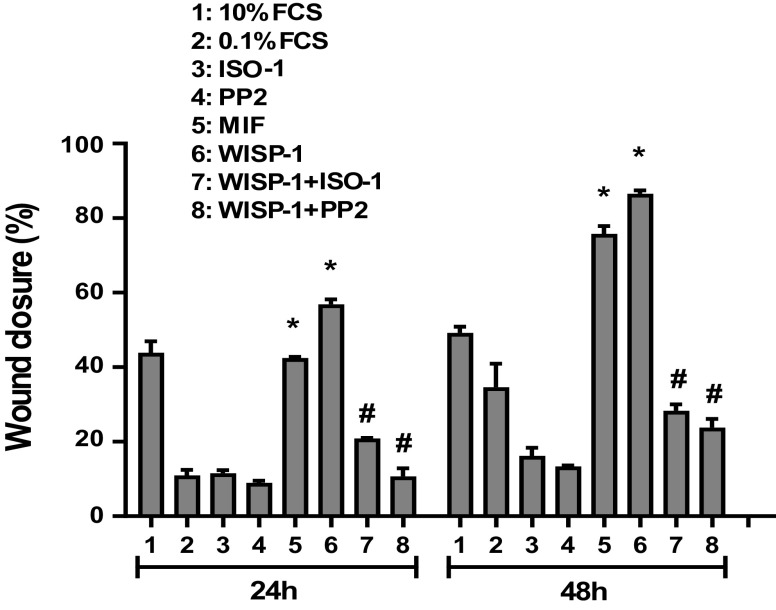
Wnt1-inducible signaling protein 1 (WISP1)**-**induced MIF promotes the migration of human lung fibroblasts (HLFs). HLFs were stimulated with migration inhibitory factor (MIF; 100 ng/mL) alone or they were pretreated for 30 minutes with the Src kinase inhibitor PP2 (1 µM) or the MIF inhibitor ISO-1 (100 μM), followed by stimulation with WISP1 (100 ng/mL) for 0, 24, and 48 h and the migration of cells was examined by the wound healing assay; 10% FCS was used as a positive control. Data represent the means area occupied by cells (%) ± SD (*n* = 3). *Statistically significant increase (*P* < 0.05) compared to control (0.1% FCS). #Statistically significant decrease (*P* < 0.05) compared to WISP1-treated cells.

The wound healing assay was used to assess the potential effects of WISP1 on HLF migration and whether MIF is implicated in these effects. As shown in Fig. 12, the migration of cells into the wounded area was significantly increased in the presence of WISP1 compared to control (0.1% FCS) 57% versus 11% (24 h) and 86% versus 34% (48 h). WISP1-induced cell migration into the wounded area was significantly decreased in the presence of PP2 and ISO-1 inhibitors, suggesting that the stimulatory effect of WISP1 is mediated by Src kinases and MIF. The latter was further supported by using rMIF since the wound closure in the presence of MIF alone was similar to that of WISP1 [42% vs. 57% (24 h) and 75% vs. 86% (48 h)].

## DISCUSSION

Considering the limited data about the factors, such as WISP1, which are involved in the expression of MIF in HLFs in inflammatory and interstitial lung diseases, our study provides further information on the regulatory role of MIF in lung inflammation and remodeling, in which fibroblasts play a central role.

We showed that WISP1 is able to stimulate the expression of MIF in HLFs in a dose- and time-dependent manner and that the WISP1-induced expression of MIF is dependent on the αvβ5-integrin. These data are in agreement with *1*) previously reported properties for WISP1, which, like the other proteins of the CCN family, is a secreted matricellular protein that does not bind to a specific receptor but signals through cell surface integrins (3, 4); and *2*) studies that report that WISP1 can stimulate the expression of IL-6 in OASMFs via integrin αvβ5 (58). Considering that the same integrin is involved in WISP1-induced expression of MIF in HLFs, it could be suggested that WISP1 induces the expression of cytokines and growth factors in fibroblasts through αvβ5-integrin, regardless of their origin. If this is the case, αvβ5-integrin could be proved to be an ideal target for the treatment of various lung diseases where fibroblasts, WISP1, and MIF play critical pathogenetic roles.

In addition to αvβ5-integrin, we also observed that WISP1-induced expression of MIF is dependent on chondroitin sulfate proteoglycans in HLFs. This finding differs from previous reports, which show that WISP1 binds decorin and biglycan carrying dermatan sulfate chains and this binding modulates its interaction with the surface of skin fibroblasts (59). Thus it appears that the type of proteoglycans mediating the effect of WISP1 on various fibroblasts may depend on the tissue origin of fibroblasts. However, even though the type of proteoglycans mediating the WISP1-induced expression of MIF is yet not clear, these chondroitin sulfate proteoglycans could be an excellent pharmacological target for various respiratory diseases in which the above molecules are implicated.

With respect to the signal transduction pathways and the identity of the factors involved in WISP1-induced expression of MIF, it was demonstrated that various factors are involved to a lesser or greater extent, among which Src kinases, PKC, NF-κB, and EGFR seem to play pivotal roles. These results are in good agreement with previous reports regarding the involvement of Src kinases and PKC in H_2_O_2_-induced MIF expression in cardiac muscle cells (63) and the activation of NF-κB by WISP1 and its involvement in WISP1-induced IL-6 expression in OASMFs (58). It is postulated that WISP1, through interaction with integrin avβ5, activates Src kinases, which in turn can activate EGFR through direct phosphorylation of its tyrosine residues, even in the absence of its ligands (transactivation). The ability of Src kinases to transactivate EGFR has been previously well documented (71, 72, 85). The exact Src kinases that are involved in the WISP1-induced expression of MIF remain to be investigated. Our observation that WISP1 induces the expression of the Src kinase Lyn, argues for the involvement of this specific kinase in WISP1-induced expression of MIF. This argument is enhanced by previous reports showing that in adenocarcinoma cells of the lung, the identified Src kinase responsible for EGFR transactivation was Lyn (73). Subsequently, the activated EGFR results in PLCγ activation by interaction, through its SH2 domains, with phosphorylated tyrosine residues in the cytoplasmic domain of EGFR (86). The activated PLCγ, in turn, triggers the phosphoionositide cascade leading to the activation of PKC. Activated PKC can then phosphorylate CARMA3 (caspase recruitment domain and membrane-associated guanylate kinase-like protein 3), ultimately leading to the activation of NF-κB, as it has been previously reported (87, 88).

**Figure 13.**
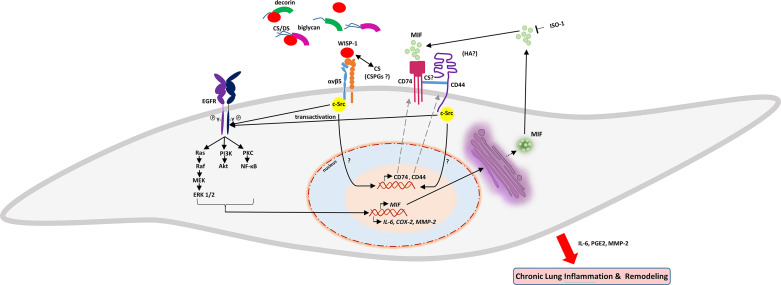
Proposed model of the effects of Wnt1-inducible signaling protein 1 (WISP1) on the expression of migration inhibitory factor (MIF) and its receptors, as well as on the expression of cyclooxygenase (COX)-2, IL-6, and matrix metalloproteinase (MMP)-2, and production of prostaglandin E_2_ (PGE_2_) from human lung fibroblasts (HLFs). The effect of WISP-1 on the expression of MIF in HLFs with the complex interactions between WISP-1, αvβ5, Src kinases, EGF receptor (EGFR), and CD74/CD44 receptors, reveal intricate regulatory networks that could contribute to the expression of proinflamamtory molecules and remodeling enzymes secretion.

Based on the data presented in this study, we propose the following model for the WISP1-induced expression of MIF in HLFs (Fig. 13). WISP1 activates αvβ5-integrin, resulting in the initiation of a doqnstream signaling cascade involving Src kinases, transactivation of EGFR, PKC activation, ultimately leading to the activation of NF-κB augmenting the expression of MIF. This proposed model is consistent with the observed experimental outcomes and provides insights into the underlying molecular events.

With respect to additional underlying molecular mechanisms, we observed that the expression of MIF was not affected in the presence of the PKA inhibitor H-89, indicating that the implication of cAMP signaling pathway in WISP1-induced expression of MIF may be mediated by the EPAC/cAMP complex and not by PKA. It is known that the EPAC/cAMP complex acting as guanine-nucleotide exchange factors for the small G proteins Rap1 and Rap2 leads to the activation of PI3K, which in turn activates Akt (89) that was found to be involved in WISP1-induced expression of MIF. Given that the expression of cyclooxygenase COX-2 is induced by WISP1, it is plausible that the activation of adenylate cyclase may be caused by PGE2, which is produced by the action of COX-2, acting through its receptors EP2 and EP4. While additional research is required to completely clarify these mechanisms, this study implies a more extensive regulatory function for WISP1. Its impact on HLFs goes beyond MIF, including the stimulation of COX-2, IL-6, and MMP-2 expression, along with the synthesis of PGE_2_ implicated in inflammation and remodeling of the ECM, processes that play significant pathogenetic roles in lung diseases (Fig. 13).

Furthermore, it should be noticed that WISP1-induced MIF mediates the stimulatory effect of WISP1 on lung fibroblast migration, which is in agreement with previous reports showing that MIF promotes the migration of human foreskin dermal fibroblasts (90).

The current study also demonstrates that WISP1 has the ability to induce the expression of CD74 and CD44, the MIF receptor and coreceptor, respectively. This finding represents a novel discovery in the existing literature. The impact of this effect is particularly significant for the coreceptor CD44, which plays a major role in the signal transduction pathways induced by MIF. CD44 is known to interact and activate Src kinases, which in turn can activate various signaling pathways of adjacent cellular receptors, including EGFR. Thus WISP1 through its ability to induce the expression of both MIF and its receptors, has the potential to enhance and amplify MIF-mediated signaling. These findings indicate that WISP1 may have a pivotal role to play in pathological conditions where MIF is involved, thereby highlighting its importance in understanding the relationship between WISP1 and these specific disease contexts.

Another important result of the present study is the finding that MIF can induce its own expression, along with the expression of its receptors CD74 and CD44. The observation that recombinant MIF is capable of inducing its expression in an autocrine manner has been previously reported in a single study involving cardiac fibroblast cultures (77). Considering that MIF itself is also able to enhance the expression of its receptors, it may be suggested that these properties of MIF may strengthen its pathogenetic role due to the continuous signaling by MIF, which can be sustained after its expression is induced by a stimulus.

It should be also noted that WISP1 and MIF through their effect on CD44, which is the main hyaluronan receptor, may be involved and influence signaling pathways in various pathological conditions in which hyaluronan is implicated.

In conclusion, the implication of WISP1 in the pathophysiology of various lung diseases may be mediated, at least in part, by the increased expression of MIF and its stimulatory effect on the production of established pathogenetic effectors. The WISP1-induced expression of MIF and relevant specific signaling pathways may be proven to be excellent candidates as novel targets to control inflammation in chronic lung diseases.

## DATA AVAILABILITY

Data will be made available upon reasonable request.

## GRANTS

This work was supported by the Hellenic Foundation for Research and Innovation (HFRI) under the 2nd Call for H.F.R.I. Scholarships to PhD Candidates (Grant 1335). The publication fees of this manuscript have been financed by the Research Council of the University of Patras.

## DISCLOSURES

No conflicts of interest, financial or otherwise, are declared by the authors.

## AUTHOR CONTRIBUTIONS

M.-E.C. and A.J.A. conceived and designed research; M.-E.C. and A.J.A. performed experiments; M.C. and A.J.A. analyzed data; M.-E.C. and A.J.A. interpreted results of experiments; M.-E.C. and A.J.A. prepared figures; M.-E.C. and A.J.A. drafted manuscript; M.-E.C., S.S.S., E.P., D.S., and A.J.A. edited and revised manuscript; M.-E.C., S.S.S., E.P., D.S., and A.J.A. approved final version of manuscript.
